# Editorial: Sensing DNA in Antiviral Innate Immunity

**DOI:** 10.3389/fimmu.2021.644310

**Published:** 2021-07-01

**Authors:** Rongtuan Lin, Junji Xing, Chunfu Zheng

**Affiliations:** ^1^ Department of Medicine, Lady Davis Institute-Jewish General Hospital, McGill University, Montreal, QC, Canada; ^2^ Immunobiology and Transplant Science Center, Department of Surgery, Houston Methodist Research Institute, Houston, TX, United States; ^3^ Department of Immunology, School of Basic Medical Sciences, Fujian Medical University, Fuzhou, China; ^4^ Department of Microbiology, Immunology and Infectious Diseases, University of Calgary, Calgary, AB, Canada

**Keywords:** DNA sensors, pattern-recognition receptors, cyclic GMP-AMP synthase, STING, viruses, innate immune responses.

The editorial team welcomes you to the specific Research Topic on “Sensing DNA in Antiviral Innate Immunity”. We appreciate the hard work and outstanding contributions from all authors. Effective defense mechanisms against virus infection and pathogenesis rely on a prompt and robust induction of antiviral innate immunity. Central to antiviral innate immune responses is the detection of evolutionarily conserved structures, termed pathogen-associated molecular patterns (PAMPs), by a set of germline-encoded pattern-recognition receptors (PRRs) (1). In the case of DNA virus or retrovirus infection, cytosolic viral DNA or reverse transcription intermediates (RTI) is detected by DNA sensors cyclic GMP-AMP synthase (cGAS) as well as other cytosolic DNA binding proteins such as interferon-gamma inducible protein 16 (IFI16) (2, 3). Conversely, endosome-associated viral nucleic acids are recognized by toll-like receptor 7 (TLR7) and TLR9 (1).

Furthermore, a recent report identified heterogeneous nuclear ribonucleoprotein A2B1 (hnRNPA2B1) as a nuclear DNA sensor for viral double-stranded DNA, but not cellular DNA, in the nucleus (4). Additionally, cGAS is recently reported to sense nuclear DNA (5–11). Following the detection of specific viral PAMPs, PRRs trigger the activation of intracellular signaling cascades, ultimately leading to the activation of NF-κB, interferon regulatory factor (IRF) 3 and 7, and the production of type I interferons (IFN-I) and various inflammatory cytokines such as CXCL10, TNFα and IL-6 (12). The antiviral program is subsequently amplified by paracrine and autocrine signaling of IFN through IFN receptors and Janus kinase (JAK)-signal transducer and activator of transcription (STAT) signaling, resulting in the induction of antiviral IFN-stimulated genes (ISGs) (13). This Research Topic will feature different contributions providing the molecular and structural basis of endosomal, nuclear, and cytosolic DNA sensors in antiviral response, the dynamic regulations of DNA sensors and their adaptor protein activation, trafficking, and post-translational modifications, and virus evasion of the host DNA-sensing antiviral innate immune responses. These approaches could contribute to the development of novel antiviral therapies and oncolytic viruses in the future.

Zahid et al. introduced the PAMPs, PRRs, and the sources of cytotoxic DNA. The authors showed the structures of endosomal and cytosolic DNA sensors alone or complexed with DNA to provide insights on how binding the DNA to these sensors triggers the signaling pathways to activate the antiviral immune responses. They focused on the endosomal DNA sensor TLR9 and multiple cytosolic DNA sensors, including cGAS, IFI16, absent in melanoma 2 (AIM2), and DNA-dependent activator IRFs (DAI). The authors also covered other cytosolic DNA sensors, including DEAD-box helicase DDX41, RNA polymerase III, DNA-dependent protein kinase (DNA-PK), and meiotic recombination 11 homolog A (MRE11), as well as adaptor protein stimulator of interferon genes (STING).

To ensure successful antiviral defenses and avoid aberrant or dysregulation of host immune signaling, antiviral pathways need to be tightly regulated. PRRs and their adaptor proteins are regulated at multiple levels. Two papers have discussed the recent advances in DNA sensors’ dynamic regulations and their adaptor protein STING. Zheng described the role of TLR9 dimerization, trafficking, and a multiprotein signaling complex formation in regulating endosomal DNA-sensing signaling. He also provided an update on the role of STING trafficking and polymerization in cGAS-STING signaling, how cellular proteins involve in cGAS-STING activation, and the evasion of cGAS-STING signaling by DNA viruses. Li et al. focused on trafficking and post-translational modifications in STING activation. They also summarized the proteins encoded by different DNA and RNA viruses to inhibit the intracellular trafficking and STING signaling activation.

Recent studies have identified nucleus-localized DNA and RNA sensors to detect pathogenic nucleic acids for triggering antiviral innate immune responses. IFI16, hnRNPA2B1, and nuclear cGAS are all defined as possible sensors for nuclear DNA that is unwinded from histone complexes. Zhang et al. summarized the recent advances in identifying nuclear DNA sensors IFI16, hnRNPA2B1, and nuclear cGAS and their roles in regulating antiviral innate immune responses and tumorigenesis. The authors also discussed the transcriptional, post-transcriptional, and post-translational regulations of these nuclear DNA sensors.

Viruses have evolved various strategies to inhibit and subvert the host’s antiviral immune responses. Three papers have been contributed to discuss recent advances in DNA viruses-induced and antagonize innate immune responses *via* DNA sensors. Zhao et al. reviewed how herpes simplex viruses (HSVs) are detected by the cytosolic DNA sensor cGAS-STING, IFI16, AIM2, and DAI, and how HSV-encoded proteins antagonize the signaling pathways triggered by DNA sensors. The authors also provided an overview of RIG-I-like receptors (RLRs) and protein kinase R (PKR) in the antiviral immune responses against HSVs. A paper from El-Jesr et al. and another paper from Lu and Zhang reviewed the latest studies on how the cytosolic DNA sensors recognize vaccinia virus (VACV) and trigger the activation of innate immune responses, and the strategies evolved by VACV to antagonize innate immune responses induced *via* cytosolic DNA sensing pathways.

In a large genome-wide association study (GWAS) of the immune responses to primary smallpox vaccination, Kennedy et al. reported a cluster of SNPs on chromosome 5 (5q31.2) that were significantly associated with IFNα response to *in vitro* poxvirus stimulation. The authors identified rs1131769, a non-synonymous SNP in *TMEM173* causing an Arg-to-His change at position 232 in the STING protein (H232 STING), as a major regulator of cGAS-mediated IFN-I production. Compared to R232 homozygote, H232 homozygote has a 90% reduction in cGAMP-mediated IFNa secretion. Molecular modeling revealed that H232 has greater structural flexibility and mobility of the ligand-binding loop.

Cytosolic DNA sensors have not been studied in livestock animals. Zheng et al. reported the characterization of porcine cGAS, STING, and IFI16 and measured the function of porcine cGAS, STING, and IFI16 in regulating IFN-I, cytokine, and ISG gene expression. Porcine cGAS-STING signaling triggers the antiviral responses, while porcine IFI16 competitively binds with agonist DNA and STING to inhibit cGAS-STING signaling. Oliveira et al. used chicken macrophage-like cell line HD11 to characterize the function of cGAS-STING signaling in chicken. Knockout studies demonstrated that chicken cGAS-STING was essential for fowlpox- and intracellular DNA-induced IFN-I responses in chicken macrophage-like cells. Furthermore, chicken cGAS-STING signaling is also required for the regulation of macrophage effector functions.

The goal of our understanding of antiviral immune responses is to develop new therapeutics. Given that viral PAMP-PRR interaction is the initial trigger of the innate and adaptive immune response, an attractive strategy for developing an efficient therapy to inhibit virus replication is natural or synthetic molecules that mimic the viral PAMPs to activate the host innate immune defense. Shao et al. reported that poly(dA:dT) treatment induced the expression of IFNs and the multiple antiviral ISGs in the cervical epithelial cells and significantly inhibited HSV-2 infection. It has been demonstrated that poly(dA:dT) can be transcribed into a 5’pppRNA in the host cells, triggering RIG-I-dependent antiviral responses. The authors showed that knockout of RIG-I significantly compromised poly(dA:dT)-mediated activation of IFN signaling and inhibition of HSV-2 infection. With an alternative approach, Abraham et al. performed a high throughput screen to identify novel small molecules capable of stimulating IFN-I production. The authors reported a small molecule termed M04 that can activate a STING-dependent IFN-I production in human cells. Mechanistically, M04 induced STING phosphorylation and ER-Golgi trafficking.

Constitutive active mutation of STING is associated with the clinical syndrome known as STING-associated vasculopathy with onset in infancy (SAVI). Berthelot et al. reviewed the similarities between T and B cell responses in severe coronavirus disease 2019 (COVID-19) and human or animal models of SAVI syndromes. The authors proposed that a delayed overactivation of cGAS-STING signaling could play a central role in COVID-19 pathogenesis. Three potential models for SARS-CoV-2 mediated STING activation were discussed.

## Author Contributions

All authors listed have made a substantial, direct, and intellectual contribution to the work and approved it for publication.

## Funding

Grant supports for this work: (1) RL lab is supported by the Canadian Institutes of Health Research grant (PJT-148657). (2) JX lab is supported by the American Heart Association Career Development Award 20CDA35260116 (Xing). (3) CZ lab is supported by the National Natural Science Foundation of China (82072263).

## Conflict of Interest

The authors declare that the research was conducted in the absence of any commercial or financial relationships that could be construed as a potential conflict of interest.
